# Brooklyn Central Dispensary

**Published:** 1856-10

**Authors:** 


*Brooklyn Central Dispensary.*—From the Annual Report just made by the trustees of this dispensary, we make the following abstract: During the year, 1,638 patients were treated—males, 716; females, 922. Adults, 1,062; children, 576. Natives of the United States, 632; Ireland, 865; England, 77; Germany, 46; Scotland, 6; Sweden, 4; Norway, 4; Denmark, 2; Canada, 1; France, 1; *Diseases*—Surgical, 503; abdomen and head, 412; chest and throat, 221; fevers, 160; skin, 76; eye and ear, 68; nervous system, 61; diseases of females, 51; pregnancy, 21; vaccination, 65. Number of patients attended at their residences, 123. Patients discharged cured or relieved, 1,555; died, 12; remaining under treatment, 71. Prescriptions gratuitously dispensed, 4,320.—*N. Y. Times.*

